# Ventilación no invasiva *versus* oxigenoterapia convencional tras fracaso de la extubación en pacientes de alto riesgo en una unidad de cuidados intensivos: un ensayo clínico pragmático

**DOI:** 10.5935/0103-507X.20210059

**Published:** 2021

**Authors:** Alberto Belenguer-Muncharaz, Maria-Lidón Mateu-Campos, Bárbara Vidal-Tegedor, María-Desamparados Ferrándiz-Sellés, Maria-Luisa Micó-Gómez, Susana Altaba-Tena, María Arlandis-Tomás, Rosa Álvaro-Sánchez, Enver Rodríguez-Martínez, Jairo Rodríguez-Portillo

**Affiliations:** 1 Unidad de Cuidados Intensivos, Hospital General Universitario de Castellón - Castelló de Plana, Spain.; 2 Unidad Predepartamental Medicina, Facultad de Ciencias de la Salud, Universitat Jaume I - Castelló de la Plana, Spain.

**Keywords:** Ventilación no invasiva, Terapia por inhalación de oxígeno, Extubación traqueal, Destete, Respiración artificial, Insuficiencia respiratoria

## Abstract

**Objetivo:**

Determinar la efectividad de la ventilación no invasiva frente a oxigenoterapia convencional en pacientes con insuficiencia respiratoria aguda tras fracaso de la extubación.

**Métodos:**

Ensayo clínico pragmático realizado una unidad de cuidados intensivos de marzo de 2009 a septiembre de 2016. Se incluyeron pacientes sometidos a ventilación mecánica > 24 horas, y que desarrollaron insuficiencia respiratoria aguda tras extubación programada, siendo asignados a ventilación no invasiva u oxigenoterapia convencional. El objetivo primario fue reducir la tasa de reintubación. Los objetivos secundarios fueron: mejora de los parámetros respiratorios, reducción de las complicaciones, de la duración de la ventilación mecánica, de la estancia en unidad de cuidados intensivos y hospitalaria, así como de la mortalidad en unidad de cuidados intensivos, hospitalaria y a los 90 días. También se analizaron los factores relacionados con la reintubación.

**Resultados:**

De un total de 2.574 pacientes, se analizaron 77 (38 en el grupo de ventilación no invasiva y 39 en el grupo de oxigenoterapia convencional). La ventilación no invasiva redujo la frecuencia respiratoria y cardíaca más rápidamente que la oxigenoterapia convencional. La reintubación fue menor en el grupo de ventilación no invasiva [12 (32%) *versus* 22(56%) en grupo oxigenoterapia convencional, RR 0,58 (IC95% 0,34 - 0,97), p = 0,039], el resto de los parámetros no mostró diferencias significativas. En el análisis multivariante, la ventilación no invasiva prevenía la reintubación [OR 0,17 (IC95% 0,05 - 0,56), p = 0,004], mientras que el fracaso hepático previo a la extubación y la incapacidad para mantener vía aérea permeable predisponían a la reintubación.

**Conclusión:**

El empleo de la ventilación no invasiva en pacientes que fracasa la extubación podría ser beneficiosa frente a la oxigenoterapia convencional.

## INTRODUCTION

The failure of extubation after mechanical ventilation (MV) has a deleterious effect, since it increases the duration of ventilation, the risk of ventilator-associated pneumonia (VAP), stay in the intensive care unit (ICU), and mortality.^(1-6)^

Noninvasive ventilation (NIV) in patients with acute respiratory failure (ARF) improves breathing and gas exchange and therefore reduces the need for intubation, shortens the hospital stay, and lowers mortality.^(7-9)^ The use of NIV in weaning after MV is indicated for support in patients at risk of extubation failure.^(7-10)^ In contrast, NIV has not shown a benefit after extubation failure; therefore, there is currently no recommendation for its use in this situation.^(9-12)^

Based on the benefits provided by NIV and despite the negative results of previous studies,^(11,12)^ the purpose of this study was to test the benefit of NIV over conventional oxygen therapy in patients who failed extubation. Our primary objective was to reduce the intubation rate. The secondary objectives were clinical improvement and reductions in complications, MV duration, ICU stay, hospital stay, and mortality in the ICU, in the hospital, and at 90 days. Factors correlated with reintubation were also analyzed.

## METHODS

A pragmatic clinical trial was conducted in a medical-surgical ICU between March 2009 and September 2016. The study was approved by the Clinical Research Ethics Committee of the *Hospital de La Plana*. Informed consent was requested from the patients or their relatives. Patients ≥ 18 years of age with medical-surgical pathology who, after a first episode of MV > 24 hours, presented ARF within 48 hours after a scheduled extubation were included. Patients who presented structural neurological disease, toxic-metabolic coma with Glasgow coma scale value < 14 during weaning or neuromuscular disease, chronic obstructive pulmonary disease (COPD), chronic respiratory disease subsidiary to receiving NIV,^(10,13-16)^ limitation of life support therapy, tracheotomy, spinal injury, scheduled surgery within the following 48 hours, or readmission or transfer to another center were excluded from the study. Patients with a contraindication to NIV were also excluded,^(7-9)^ although an NIV trial was performed in patients with excess of secretions or postextubation stridor.^(9)^

### Protocol

Weaning was considered to begin in conscious patients (Glasgow coma scale 14 - 15 points) when they had MV in pressure support ventilation mode, a fraction of inspired oxygen (FiO_2_) ≤ 0.5, positive end-expiratory pressure (PEEP) + 5cmH_2_O, noradrenaline ≤ 0.2mcgr/kg/min, temperature <38°C, and an absence of acidosis. The weaning process consisted of a spontaneous breathing trial, which is usually performed in our unit through a T-tube,^(1,17)^ which all patients in the study performed. The T-tube trial was considered successful according to the established guidelines after 30 - 120 minutes.^(18,19)^ During one nursing shift (8 hours), the number of times secretions were aspirated (none, one, two or more), as well as the cough capacity (capacity of the mucus to reach the orotracheal tube), were recorded before the last T-tube trial. Once the trial was passed successfully, extubation and subsequent placement of a Venturi mask were performed (FiO_2_ 0.3 - 0.4). If T-tube was not passed,^(18)^ the patient was reconnected to the ventilator in pressure support ventilation mode for later performance of the T-tube trial on successive days. The final decision of extubation or reconnection was made by the responsible physician. Patients with ARF during the 48 hours following extubation were evaluated for inclusion in the study by the attending physician. Extubation failure was considered when the following was observed: use of accessory muscles, paradoxical breathing, respiratory rate (RR) > 25bpm or an increase greater than 50% over baseline for 2 hours, together with gasometric deterioration [partial pressure of oxygen - PaO_2_ < 65mmHg or partial pressure of carbon dioxide (PaCO_2_) > 45mmHg (pH < 7.33)].^(19)^ Extubation failure was classified as^(20)^ a) airway pathology: postextubation stridor, excess secretions; b) pathology without airway involvement: pulmonary edema, hypoxemic and/or hypercapnic ARF, or encephalopathy. Patients who required urgent intubation within 48 hours after extubation were not included in the study. Lastly, patients excluded by the physician’s decision were not included.

After being deemed eligible for inclusion, each patient was assigned to a group, the study group (NIV) or the control group (conventional oxygen therapy), by opening a sealed envelope given them by the attending physician. The simple randomization was carried out before the study began by a physician not belonging to the study, using a computerized system.

### Noninvasive ventilation

The BiPAP® Vision (Respironics Inc., Murrysville, PA, USA) was used with oronasal and facial masks (Total face® and PerforMax®, respectively) (Respironics Inc., Murrysville, PA, USA), along with an active humidification system (MR850, Fischer & Payckel, Auckland, New Zealand). In addition, continuous positive airway pressure (CPAP) from a Boussignac valve (Vygon(, Ecouen, France) was delivered through an oronasal mask. Procedure:^(8)^ Once the patient was informed about the procedure, the type of mask was selected according to their anatomy, and the harness was placed. In the case of NIV, ventilation was initiated with progressive levels of inspiratory positive airway pressure (IPAP) and expiratory positive airway pressure (EPAP) until a minimum IPAP of 10 - 15cmH_2_O and an EPAP of 5 - 6cmH_2_O were reached in the first hour of support. In CPAP, the minimum initial PEEP level was 5cmH_2_O, with progressive increases up to 10 - 15cmH_2_O. The objective of pressures of both devices was to reduce dyspnea, the use of accessory muscles, paradoxical breathing, and RR. The FiO_2_ from both devices was adjusted to obtain a oxygen saturation pulse oximeter (SaO_2_) of 94 - 96%. After adaptation of the mask, it was adjusted to the face of the patient using adjustable straps.

### Conventional oxygen therapy

The control group received oxygen therapy through a Venturi mask (FiO_2_ of 0.5) or a non-rebreather mask connected to a high-flow flowmeter set to 30L/min O_2_ (estimated FiO_2_ of 1.0).

Both NIV/CPAP and oxygen therapy were maintained continuously until the patient experienced clinical and/or gasometric improvement. In the NIV/CPAP patients, pressure levels were progressively reduced until complete disconnection, at which time they were switched to a Venturi mask (FiO_2_ 0.3 - 0.4). No need to reinstate such support due to clinical worsening in the following 48 hours after withdrawal of it was considered successful. Failure and indications of intubation followed established criteria in both groups.^(9)^ The modifications of FiO_2_ and levels of IPAP/EPAP or PEEP, as well as the time of orotracheal intubation, were performed according to the criteria set by the responsible physician. Patients received aspiration of secretions, postural changes, incentive spirometry, and bronchodilators at the discretion of the physician.

After inclusion in the study, demographic data, the cause of MV,^(21)^ severity measured using the Simplified Acute Physiological Score (SAPS) 3, organ failure scale using the Sequential Organ Failure Assessment (SOFA)^(22)^ (both at ICU admission), and comorbidities were recorded. Before extubation, the worst value of organ failure by SOFA was recorded, as was the type and duration of each sedative, analgesic, and neuromuscular blocking agents used. The duration from MV to first extubation, from the start of weaning to extubation, and from the last T-test, as well as the time from extubation to failure, were calculated. Hemodynamic variables (mean arterial pressure - MAP, HR), respiratory (RR, SaO_2_), and blood gas levels were collected at the time of extubation failure. Likewise, RR and HR were collected during the 1st, 2nd, and 8th hours after randomization to analyze the clinical improvement as estimated by the reductions in both parameters. After extubation failure, the following variables were collected: need for reintubation, tracheotomy, infections (pneumonia or tracheobronchitis associated with MV, urinary tract infection, bacteremia),^(23)^ organ failure after allocation using the SOFA scale,^(22)^ need for dialysis, need for surgery, and need for NIV or reintubation (both after the study period). The duration of the first MV period (until withdrawal of any of the devices under study), the duration of NIV or conventional oxygen therapy [time from allocation to withdrawal of ventilatory support (in the NIV group) and transition to Venturi mask, or a reduction in FiO_2_ ≤ 0.4 (in the conventional oxygen therapy group)] was calculated, as were the overall duration of MV (considered complete withdrawal of any ventilation device or stoppage of high-concentration oxygen therapy), the ICU stay, and the hospital stay. Mortality in the ICU, in the hospital, and at 90 days was collected.

### Statistical analysis

Based on previous results,^(11,24)^ we thought that the need for intubation could be reduced by 32% (69% in the conventional oxygen therapy group *versus* 37% in the NIV group). The estimated sample needed was 35 subjects in each group, with a 95% confidence interval - 95%CI (1-α) and a power of 80%. The statistical tests used for quantitative variables were Student’s t-test or the Mann-Whitney U test, according to the normality of each variable. For qualitative variables, the chi-squared test was used with Fisher’s exact test. Differences were considered significant if p < 0.05. The relative risk (95%CI) of the variables under study and Cox regression for mortality at 90 days (together with the Kaplan-Meier cumulative survival) were calculated. The analysis was performed by intention-to-treat. With the aim of analyzing the influence of both groups on RR and HR, a multivariate analysis (with Bonferroni correction) of repeated samples was performed. A multivariate binary logistic regression analysis of the predictors of reintubation was performed, and the influence of NIV to avoid reintubation was analyzed. The inability to maintain airway patency was included,^(25)^ as were those variables that were significant (p ≤ 0.05) before extubation failure (smoking, hepatic, renal, hemodynamic or hematological failure) plus the use of NIV or oxygen therapy. The data were analyzed in the statistical package SPSS 20.0.

## RESULTS

**Figure 1:**
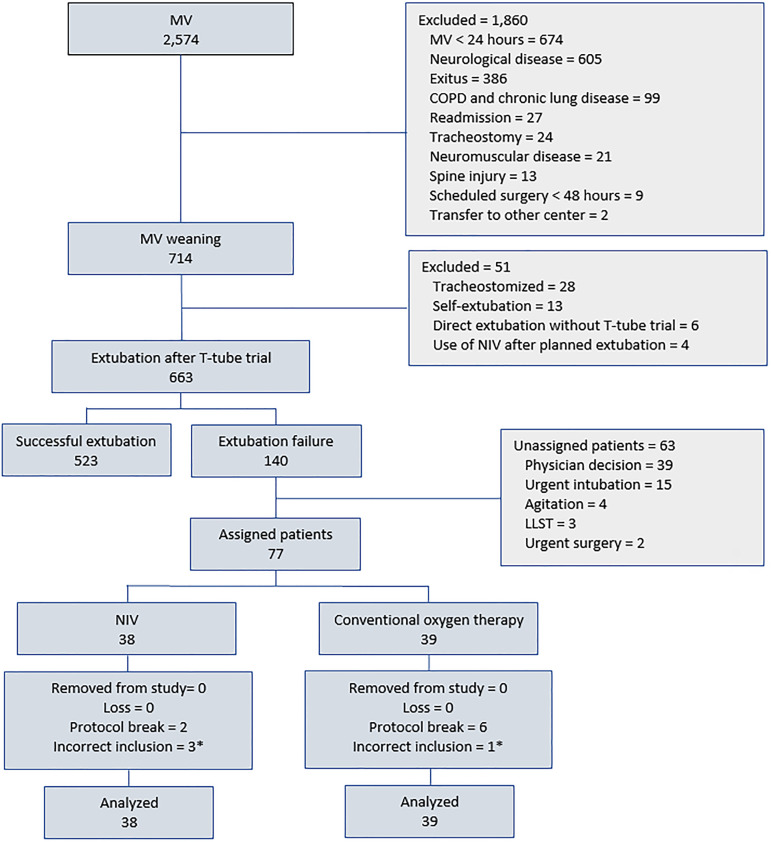
Flow diagram. MV - mechanical ventilation; COPD - chronic obstructive pulmonary disease; LLST - limitation of life support therapy; NIV - noninvasive ventilation. * Incorrect inclusion: chronic obstructive pulmonary disease (two patients in the noninvasive ventilation group, one in the conventional oxygen therapy group), neuromuscular (one patient in the noninvasive ventilation group).

During the study period, a total of 2,574 patients (Figure 1) were analyzed, of whom 663 were extubated on a scheduled basis. In 140 (21%) patients, extubation failed, and 77 were finally assigned. Sixty-three patients were not randomized for various reasons (39 by facultative decision and 15 by urgent intubation). After the trial, there were eight protocol breaks and four incorrect randomizations because they met one or more exclusion criteria, all of which were included in the final analysis (38 in NIV and 39 in conventional oxygen therapy).

As shown in table 1, the majority of the sample was men, with an average age of > 60 years, who received MV mainly for ARF. As a sedative, a combination of propofol and midazolam was most often used. The median duration of MV was 13 - 14 days, and that of weaning was 4 days. Most patients were extubated after passing their first T-tube trial. The baseline characteristics at inclusion did not show significant differences, except for a higher percentage of smoking in the control group (44% *versus* 18% in the NIV group, p = 0.026). The main cause of extubation failure was ARF unrelated to airway management (74% in NIV *versus* 59% in control). There were no differences in the causes of extubation failure or in the clinical-gasometric variables at the time of failure or at the time of randomization (Table 2).

**Table 1:** Demographic characteristics, comorbidities, and clinical parameters during the period of weaning from mechanical ventilation

	NIV (n = 38)	Conventional oxygen therapy (n = 39)
Sex, male	19 (50)	22 (56)
Age, years	66 (58 - 76)	62 (49 - 73)
BMI (kg/m^2^)	29 ± 7	27 ± 6
SOFA at ICU admission	2 (1 - 2)	2 (1 - 2)
SAPS 3 at ICU admission	56 (51 - 67)	58 (55 - 67)
Comorbidities		
Hypertension	21 (55)	17 (47)
Diabetes mellitus	13 (34)	10 (26)
Chronic renal failure	7 (18)	3 (8)
Chronic heart failure	4 (10)	3 (8)
Obstructive sleep apnea	2 (5)	1 (3)
Smoking	7 (18)	17 (44)
Alcohol	4 (10)	8 (20)
Cause of mechanical ventilation		
ARF*	25 (66)	24 (61)
Postoperative	12 (32)	12 (31)
Coma	1 (2)	1 (8)
Sedatives during mechanical ventilation (n = 54)		
None	1/27 (4)	1/27 (4)
Propofol	8/27 (30)	8/27 (30)
Midazolam	5/27 (18)	5/27 (18)
Propofol and midazolam	13/27 (48)	13/27 (48)
Morphine	25/27 (93)	26/27 (96)
Cisatracurium	2/27 (7)	2/26 (8)
Propofol (days)	4 (2 - 5)	5 (3 - 7)
Midazolam (days)	8 (4 - 14)	9 (5 - 13)
Parameters for weaning from mechanical ventilation		
Time from onset of MV to extubation (days)	13 (4 - 19)	14 (10 - 24)
Start time weaning to extubation (days)	4 (2 - 7)	4 (2 - 10)
Number of aspirations before the last T-tube trial (n = 68)		
None	1/32 (3)	3/36 (8)
1 aspiration	17/32 (53)	17/36 (47)
2 aspirations	9/32 (28)	7/36 (19)
≥ 3 aspirations	5/32 (15)	9/36 (25)
Strength to cough (n = 68)	19/33 (58)	25/35 (71)
Duration of last T-tube trial (hours)	2 (1 - 3)	2 (2 - 4)
Extubation in the first T-tube trial	27 (71)	21 (54)

NIV - noninvasive ventilation; BMI - body mass index; SOFA - Sequential Organ Failure Assessment; ICU - intensive care unit; SAPS - Simplified Acute Physiology Score; ARF - acute respiratory failure; MV - mechanical ventilation.

* Causes of acute respiratory failure in the noninvasive ventilation group (n = 25): pneumonia (n = 6), sepsis (n = 4), cardiorespiratory arrest (n = 5), acute postoperative respiratory failure (n = 2), acute edema of cardiogenic lung (n = 4), trauma (n = 2), bronchoaspiration (n = 2). Causes of acute respiratory failure in the conventional oxygen therapy group (n = 24): pneumonia (n = 6), sepsis (n = 3), cardiorespiratory arrest (n = 4), acute postoperative respiratory failure (n = 3), acute edema of cardiogenic lung (n = 2), trauma (n = 2), acute respiratory distress syndrome (n = 2), bronchoaspiration (n = 2). Results expressed as n (%), median and interquartile range (25-75) or mean ± standard deviation.

**Table 2:** Cause of extubation failure and the hemodynamic and respiratory parameters at the time of randomization

	NIV (n = 38)	Conventional oxygen therapy (n = 39)
Time from extubation to postextubation ARF (hours)	7 (2 - 18)	5 (1 - 28)
Cause of extubation failure		
ARF not related to airway*	28 (74)	23 (59)
Inability to maintain airway patency†	10 (26)	16 (41)
Clinical parameters at the time of ARF		
Respiratory rate > 25bpm	30 (79)	30 (77)
RR increase > 50% with respect to baseline	23 (60)	22 (56)
PaO_2_ < 65 mmHg	19 (50)	18 (46)
PaCO_2_ > 45 mmHg	14 (37)	15 (38)
pH < 7.33	18 (48)	18 (46)
PaO_2_/FiO_2_ < 250	23 (60)	26 (67)
Work of breathing	32 (84)	30 (77)
Mean arterial pressure (mmHg)	94 ± 18	97 ± 18
Heart rate (bpm)	107 ± 21	101 ± 25
Respiratory rate (bpm)	32 ± 9	33 ± 10
pH (mmHg)	7.36 ± 0.11	7.38 ± 0.10
PaCO_2_ (mmHg)	48 ± 25	53 ± 63
PaO_2_/FiO_2_	187 ± 86	149 ± 59
Lactate (mmol/L)	1 ± 1	1 ± 2

NIV - noninvasive ventilation; ARF - acute respiratory failure; RR - respiratory rate; PaO_2_ - arterial oxygen pressure; PaCO_2_ - partial pressure of carbon dioxide; FiO_2_ - fraction of inspired oxygen.

* Causes of acute respiratory failure not related to the airways: NIV group: acute respiratory failure (n = 22), acute cardiogenic lung edema (n = 5), encephalopathy (n = 1); conventional oxygen therapy group: acute respiratory failure (n = 20), acute cardiogenic lung edema (n = 3); † causes of acute respiratory failure related to the airways: noninvasive ventilation group: poor management of secretions (n = 8), laryngomalacia (n = 2); conventional oxygen therapy group: poor management of secretions (n = 12), laryngomalacia (n = 4). Results expressed as median and interquartile range (25 - 75), n (%) or mean ± standard deviation.

**Figure 2:**
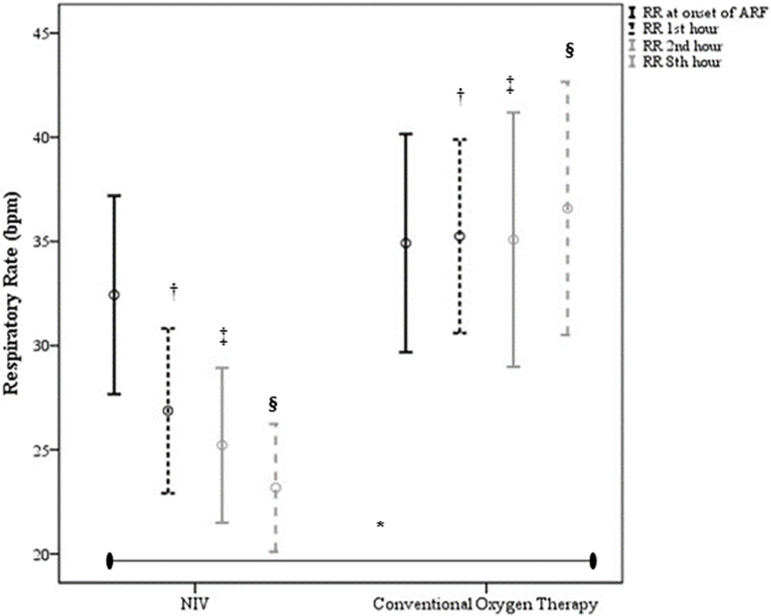
Evolution of respiratory rate comparing noninvasive ventilation (n = 23) *versus* conventional oxygen therapy (n = 12). NIV - noninvasive ventilation; RR - respiratory rate; ARF - acute respiratory failure. Bonferroni correction * noninvasive ventilation *versus* conventional oxygen therapy (p = 0.003); † noninvasive ventilation *versus* conventional oxygen therapy in the 1st hour (p = 0.01); ‡ noninvasive ventilation *versus* conventional oxygen therapy in the 2nd hour (p = 0.004); § noninvasive ventilation *versus* conventional oxygen therapy in the 8th hour (p = 0.0001).

**Figure 3:**
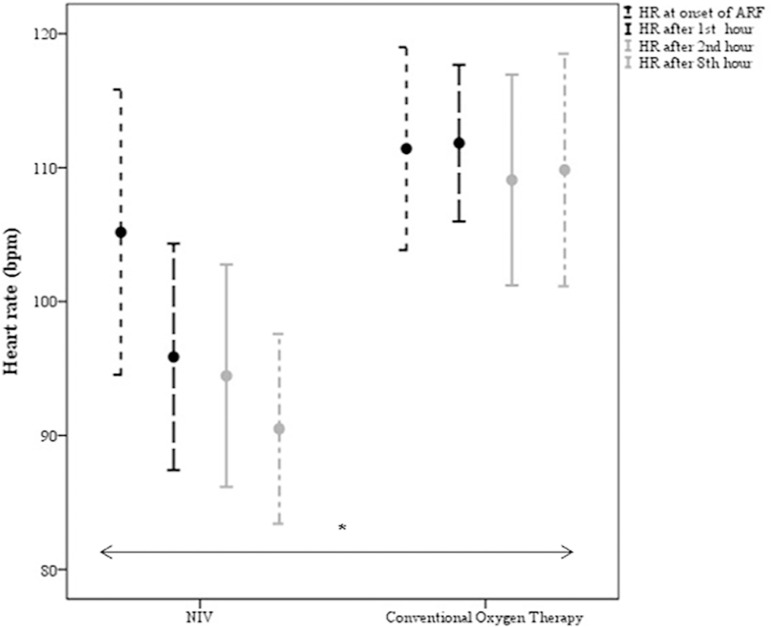
Evolution of heart rate comparing noninvasive ventilation (n = 22) *versus* conventional oxygen therapy (n = 12). NIV - noninvasive ventilation; HR - heart rate; ARF - acute respiratory failure. * Noninvasive ventilation *versus* conventional oxygen therapy (p = 0.016).

In the study group, NIV was used in 36 patients, and CPAP was used in two patients. The pressures used in NIV and CPAP in the first hour were IPAP 16 ± 5cmH_2_O, EPAP 6 ± 2cmH_2_O, and PEEP = 5cmH_2_O. FiO_2_ in the first hour did not show significant differences (0.54 ± 0.2 in the NIV group *versus* 0.56 ± 0.2 in the control group). In the first 8 hours of follow-up, significant reductions in RR (Figure 2) and HR (Figure 3) were observed in the NIV group *versus* the control group [(p = 0.003) and (p = 0.016), respectively].

**Figure 4:**
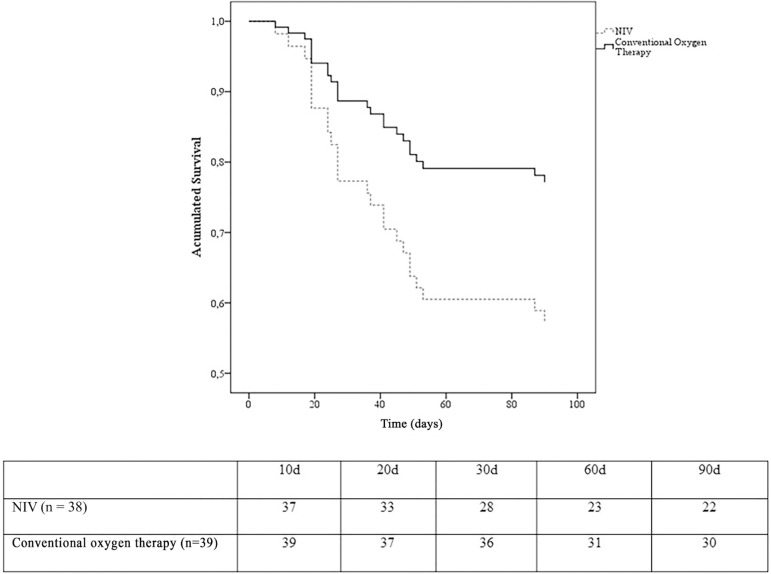
Kaplan-Meier survival analysis comparing noninvasive ventilation *versus* conventional oxygen therapy at 90 days. NIV - noninvasive ventilation. Log rank test (p = 0.068). The table shows the number of subjects who survived during the study period.

**Figure 5:**
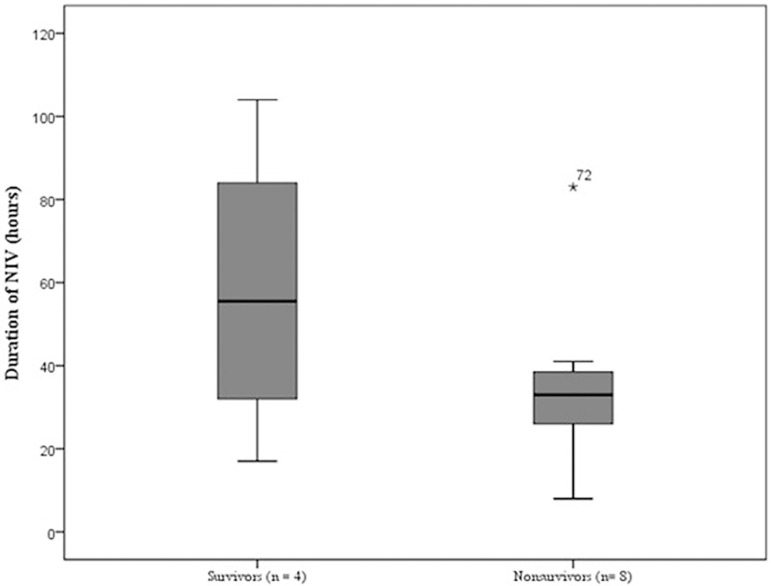
Comparison of the duration of noninvasive ventilation between survivors and nonsurvivors who required orotracheal intubation. NIV - noninvasive ventilation. p = 0.315.

Regarding the primary objective (Table 3), a lower percentage of reintubation was observed in the NIV group [12 (32%) *versus* 22 (56%) in the conventional oxygen therapy group, relative risk 0.58 (95%CI 0.34-0.97), p = 0.039]. In both groups, 50% of patients were reintubated for problems related to the airways (mainly due to poor management of secretions). The duration of support after extubation failure was greater in the NIV group [36 (20 - 79) hours *versus* 14 (3 - 39) hours in conventional oxygen therapy, p = 0.003]. Among the rest of the variables analyzed, a higher rate of complications, a longer duration of MV, and longer ICU and hospital stays were observed in the control group, without reaching significance. There were no significant differences in mortality at ICU discharge, at hospital discharge, or at 90 days (Table 3 and Figure 4). Nine (75%) of the 12 intubated patients in the NIV group developed multiorgan failure, causing their death (100%). The duration of ventilation within the NIV failure group was similar between survivors and nonsurvivors (Figure 5).

**Table 3:** Analysis of primary and secondary objectives achieved after extubation failure

	NIV (n = 38)	Conventional oxygen therapy (n = 39)	p value	Relative risk (95%CI)
Reintubation	12 (32)	22 (56)	0.039*	0.58 (0.34 - 0.97)
Tracheotomy	7 (18)	10 (26)	0.584*	0.79 (0.42 - 1.47)
Tracheobronchitis or VAP†	4 (10)	8 (20)	0.347*	0.63 (0.27 - 1.46)
Urinary tract infection‡	7 (18)	10 (26)	0.584*	0.79 (0.42 - 1.47)
Bacteremia§	7 (18)	3 (8)	0.309*	1.49 (0.92 - 2.40)
Hemodynamic failure	11 (29)	11 (28)	1.000*	1.01 (0.62 - 1.67)
Acute renal failure	13 (34)	11 (28)	0.628*	1.14 (0.72 - 1.82)
Hepatic failure	6 (16)	1 (3)	0.056*	1,87 (1.26 - 2.78)
Renal replacement therapy	4 (10)	4 (10)	1.000*	1.01 (0.48 - 2.11)
Reintubation after 48 hours	3 (8)	4 (10)	1.000*	0.85 (0.35 - 2.08)
NIV after 48 hours	2 (5)	2(5)	1.000*	1.01 (0.37 - 2.77)
Surgery after extubation failure	1 (3)	3 (8)	0.615*	0.49 (0.08 - 2.73)
Duration of NIV or conventional oxygen therapy (hours)	36 (20 - 79)	14 (3 - 39)	0.003	
Duration of first episode of MV (days)	12 (5 - 20)	14 (9 - 24)	0.165	
Overall duration of MV¶ (days)	14 (7 - 22)	14 (7 - 29)	0.303	
ICU stay (days)	17 (10 - 30)	27 (14 - 36)	0.219	
Hospital stay (days)	39 (23 - 57)	45 (31 - 58)	0.347	
Multiorgan failure during evolution	9 (24)	7 (18)	0.579*	1.18 (0.71 - 1.96)
Causes of multiorgan failure				
Septic shock||	2	3		
Decompensation of liver cirrhosis	1	1		
Hemorrhagic shock	0	1		
Refractory heart failure	2	0		
Maintained MOD#	4	2		
Mortality in ICU	9 (24)	6 (15)	0.404	1.28 (0.78 - 2.09)
Mortality 90-d**	16 (42)	9 (23)	0.068	2.14 (0.94 - 4.85)
Hospital mortality	16 (42)	9 (23)	0.092	1.51 (0.98 - 2.33)

NIV - noninvasive ventilation; 95%CI - 95% confidence interval; VAP - ventilator-associated pneumonia; MV - mechanical ventilation; ICU - intensive care unit; MOD - multiorgan dysfunction. * Fisher's exact test; † causes of tracheobronchitis or ventilator-associated pneumonia: noninvasive ventilation group: *Pseudomonas aeruginosa* (n = 3), methicillin-sensitive *Staphylococcus aureus* (n = 1); conventional oxygen therapy group: *P. aeruginosa* (n = 4), *Escherichia coli* (n = 2), *Klebsiella pneumoniae* (n = 1), methicillin-sensitive *S. aureus* (n = 1)]; ‡ causes of urinary tract infection: noninvasive ventilation group: *Candida albicans* (n = 3), *E. coli* (n = 2), *Pseudomonas aeruginosa* (n = 1), *Candida tropicalis* (n = 1)]; conventional oxygen therapy group: *E. coli* (n = 2), *C. albicans* (n = 2), one case each of *Enterococcus faecalis, Candida parapsilosis, Klebsiella* ESBL, *E. coli* ESBL, and *Staphylococcus hominis*; § causes of bacteremia: noninvasive ventilation group: *Staphylococcus epidermidis* (n = 4), *P. aeruginosa* (n = 2), *K. pneumoniae* (n = 1)]; conventional oxygen therapy group: *Staphylococcus epidermidis* (n = 2), *E. coli* (n = 1); ¶ adding invasive and noninvasive ventilation until complete disconnection of mechanical ventilation; || causes of septic shock: noninvasive ventilation (n = 2): mesenteric ischemia (n = 1) and intestinal perforation (n = 1); conventional oxygen therapy (n = 3): mesenteric ischemia (n = 1), unknown cause (n = 2); # evolution of prolonged multiorgan dysfunction during ICU stay; ** mortality at 90 days measured by Cox regression. Results expressed as n (%) or median and interquartile range.

The analysis of the factors related to reintubation showed that the inability to maintain airway patency as a cause of extubation failure and the presence of hepatic failure (measured by SOFA) before extubation were determinants for reintubation. In contrast, the use of NIV prevented reintubation [odds ratio 0.17 (95%CI 0.05 - 0.56), p = 0.004] (Table 4).

**Table 4:** Analysis of factors related to the need for reintubation after extubation failure

	Number of OTI patients / number of patients (%)	Univariate analysis	p value	Multivariate analysis	p value
		RR (95%CI)		RR (95%CI)	
NIV *versus* conventional oxygen therapy (n = 77)					
NIV	12/38 (32)	0.56 (0.32 - 0.96)	0.03	0.17 (0.05 - 0.56)	0.004
Conventional oxygen therapy	22/39 (56)				
Smoking (n = 77)					
Yes	13/24 (54)	1.36 (0.83 - 2.24)	0.32		
No	21/53 (40)				
Liver failure before extubation (n = 74)					
Yes	8/9 (89)	2.22 (1.52 - 3.23)	0.01	16.31 (1.50 - 176.67)	0.005
No	26/65 (40)				
Renal failure before extubation (n = 74)					
Yes	21/35 (60)	1.80 (1.07 - 3.02)	0.03	2.94 (0.85 - 10.11)	0.087
No	13/39 (33)				
Hemodynamic failure before extubation (n = 73)					
Yes	25/48 (52)	1.44 (0.80 - 2.60)	0.22		
No	9/25 (36)				
Hematological failure before extubation (n = 73)					
Yes	12/17 (71)	1.79 (1.14 - 2.81)	0.02	1.38 (0.34 - 5.50)	0.648
No	22/56 (39)				
Failure of extubation due to inability to maintain airway patency (n = 77)					
Yes	15/24 (62)	1.74 (1.08 - 2.80)	0.04	5.14 (1.44 - 18.36)	0.012
No	19/53 (36)				

OTI - orotracheal intubation; RR - relative risk; 95%CI - 95% confidence interval; NIV - noninvasive ventilation.

## DISCUSSION

Noninvasive ventilation reduced the rate of reintubation after extubation failure, as well as in the rest of the target variables. In the multivariate analysis, NIV protected against reintubation. Until now, all studies had questioned its usefulness.^(10-12)^ Therefore, the results obtained in another study on daily clinical practice are relevant: Many of its participants had a high risk of extubation failure [> 65 years, overweight, previous cardiac pathology, prolonged MV (> 7 days) due to pneumonia, sepsis or cardiorespiratory arrest, and many secretions].^(26)^

After the removal of the positive pressure generated by the MV, changes in the airway or in the cardiorespiratory system (including muscle function) that can lead to extubation failure often occur.^(26)^ As in our study, the most frequent causes of extubation failure are respiratory failure (65%) and the inability to protect the airway (10-20%).^(6)^ In respiratory failure (due to diaphragmatic weakness, fluid overload, or heart failure), the application of positive pressure (IPAP and EPAP) can be beneficial. Inspiratory positive airway pressure can provide support to the respiratory muscles (mainly the diaphragm), reducing energy expenditure, and EPAP/PEEP can act at two levels: 1) by increasing functional residual capacity, tidal volume, and oxygenation; and 2) by conditioning a reduction in preload in both ventricles and in the afterload of the left ventricle.^(26-28)^ We observed an important physiological response to NIV: a reduction in RR and HR with respect to those in the control group. Likewise, a small observational study showed improved respiratory parameters (RR, tidal volume) and blood gas, as well as a decrease in oxygen consumption and energy expenditure, after extubation failure when they used NIV and CPAP compared to oxygen therapy.^(29)^ In contrast, in the subgroup of patients who could not maintain airway patency, NIV was not effective, given the high rate of intubation observed (75%) and because it is a predictor of reintubation. We believe that the ability to maintain airway patency should be routinely assessed (cuff-leak test^(26,30)^ and secretion score^(26,31)^) together with respiratory trials^(32)^ to evaluate the need for NIV and respiratory physiotherapy after extubation. The benefit of NIV in patients with little ability to maintain airway patency (estimated by a peak cough flow < 70L/min) was reflected in an observational study, where it reduced the intubation rate compared to the control treatment (9% *versus* 35%) at 72 hours after extubation, p < 0.01).^(33)^ These results would support the use of NIV together with the aforementioned measures.

The NIV failure rate in observational studies ranges from 13% to 38%;^(24,25,29,34)^ in contrast, the failure rate has been higher in clinical trials, between 48% and 72%.^(11,12)^ The main characteristics of our study, which could explain the different results, are the following: First, we did not include COPD patients, given the benefit of NIV to them.^(10,13,15,16)^ Second, in the study by Esteban et al.,^(12)^ NIV rescue was investigated in patients in the control group (n = 28), where an NIV failure rate of 25% was observed, in line with the results of observational studies.^(24,25,29,34)^ Third, the levels of IPAP/EPAP in the study by Keenan et al.^(11)^ were lower (IPAP 10 ± 2cmH_2_O, EPAP 5 ± 1cmH_2_O) than those used here (IPAP 16 ± 5cmH_2_O, EPAP 6 ± 2cmH_2_O). In various studies, the main cause of reintubation is the persistence of dyspnea as a sign of muscle fatigue.^(4,12)^ Therefore, it would be necessary to provide an adequate pressure level (> 15cmH_2_O pressure support) that can reduce muscle fatigue and dyspnea in order to avoid reintubation.^(28,29)^ The levels of IPAP used in our study are in line with those recommended,^(27,28)^ which could have influenced the results obtained.

Surprisingly, despite the reduction in the intubation rate in the NIV group, a nonsignificant reduction was observed in the rest of the objectives analyzed. The shorter duration of conventional oxygen therapy stands out, probably due to the failure to control the signs of respiratory fatigue, as shown in figures 2 and 3, which led to earlier intubation. The longer duration of support in NIV, the presence of complications after the study period, and the small sample size may have made the improvements in the NIV group not as evident.

Like various studies,^(12,35)^ this study observed an increase in mortality related to NIV failure, which was striking at 90 days and at hospital discharge. Perhaps more than the failure of NIV as a mortality factor, it could be the high number of patients who developed multiorgan failure due to complications associated with their underlying pathology (a fact also observed in the oxygen therapy group) that led to death. This theory would be supported by the long time elapsed from randomization to death of most of the nonsurvivors (20 - 30 days). We believe that these factors were related to mortality in the ICU, where the differences in mortality were centered on three patients (24% *versus* 15%); on the other hand, mortality in the hospital would not be influenced by the device used. Along these lines, an editorial that analyzed the results of a clinical trial^(12)^ showed that NIV success [relative risk 1.66 (95%CI 0.51 - 5.37)] or NIV failure did not influence mortality [relative risk 1.77 (95%CI 0.95 - 3.30)].^(36)^ Likewise, an observational study found no increase in mortality associated with failure after the use of NIV (29% *versus* 27% without NIV, p = 0.77).^(4)^ Another factor that has been correlated with mortality is the prolongation of ventilation in those patients who have failed NIV.^(12,35)^ In contrast, we did not verify this relationship, nor did two other observational studies in hypoxemic patients, observed similar numbers of complications at the time of intubation^(37)^ and similar mortality rates.^(38)^

Regarding the predictive factors of reintubation, we found that the inability to maintain airway patency and a previous decompensated liver disease were determinants of reintubation. As we pointed out at the beginning of the Discussion, the role of NIV in the inability to maintain airway patency has yet to be determined; therefore, we should expect the failure of NIV in patency-failure patients. In contrast, NIV proved beneficial over oxygen therapy as a means to prevent reintubation, which would answer the question that drove this study.

The role of high-flow nasal oxygen therapy (HFNOT) has been relevant in recent years. Although its use as support in weaning has been studied, its efficacy in subjects who fail extubation has not yet been proven.^(39)^ A recent clinical trial supports the use of NIV together with HFNOT *versus* HFNOT alone to avoid extubation failure in patients at risk.^(40)^ At the time of this study, HFNOT was not available in our center.

The main weaknesses of this study are the long period of patient enrollment due to its being a single-center study with strict exclusion criteria, the low failure rate probably due to the prolongation of MV, and, finally, the use of NIV right after extubation failure in candidates who were not included in the study at the discretion of the attending physician. This last subset of patients would have had a faster inclusion, which would have shortened the timeframe of the study. On the other hand, the high rate of respiratory failure not related to the airways in the NIV group could have influenced the findings of the superiority of NIV over oxygen therapy. Protocol breaks (six in the oxygen therapy group) could also have influenced the results in favor of NIV, and could the low use of CPAP.

## CONCLUSION

Noninvasive ventilation in patients who fail extubation could be beneficial compared to conventional oxygen therapy.
